# Recovery and functional outcome after radial nerve palsy in adults with a humeral shaft fracture: a multicenter prospective case series

**DOI:** 10.1016/j.jseint.2023.02.003

**Published:** 2023-02-23

**Authors:** Saskia H. Van Bergen, Esther M.M. Van Lieshout, Michael H.J. Verhofstad, Dennis Den Hartog, HUMMER Investigators, Ivo Beetz, Hugo W. Bolhuis, P. Koen Bos, Maarten W.G.A. Bronkhorst, Milko M.M. Bruijninckx, Jeroen De Haan, Axel R. Deenik, P. Ted Den Hoed, Martin G. Eversdijk, J. Carel Goslings, Robert Haverlag, Martin J. Heetveld, Albertus J.H. Kerver, Karel A. Kolkman, Peter A. Leenhouts, Kiran C. Mahabier, Sven A.G. Meylaerts, Ron Onstenk, Martijn Poeze, Rudolf W. Poolman, Bas J. Punt, Ewan D. Ritchie, W. Herbert Roerdink, Gert R. Roukema, Jan Bernard Sintenie, Nicolaj M.R. Soesman, Edgar J.T. Ten Holder, Wim E. Tuinebreijer, Maarten Van der Elst, Frank H.W.M. Van der Heijden, Frits M. Van der Linden, Peer Van der Zwaal, Jan P. Van Dijk, Hans-Peter W. Van Jonbergen, Egbert J.M.M. Verleisdonk, Jos P.A.M. Vroemen, Marco Waleboer, Philippe Wittich, Wietse P. Zuidema

**Affiliations:** a Trauma Research Unit, Department of Surgery, Erasmus MC, University Medical Center Rotterdam, Rotterdam, The Netherlands; b Department of Surgery, Gelre Hospital, Apeldoorn, The Netherlands; c Department of Orthopaedic Surgery, Erasmus MC, University Medical Center Rotterdam, Rotterdam, The Netherlands; d Trauma Unit, Haaglanden MC, The Hague, The Netherlands; e Department of Surgery, IJsselland Hospital, Capelle a/d Ijssel, The Netherlands; f Department of Surgery, Dijklander Ziekenhuis, Hoorn, The Netherlands; g Department of Orthopaedic Surgery, Haaglanden MC, The Hague, The Netherlands; h Department of Surgery, Ikazia Hospital, Rotterdam, The Netherlands; i Department of Surgery, St. Jansdal Hospital, Harderwijk, The Netherlands; j Trauma Unit, Department of Surgery, Amsterdam University Medical Center, location AMC, Amsterdam, The Netherlands; k Department of Surgery, OLVG, Amsterdam, The Netherlands; l Department of Surgery, Spaarne Gasthuis, Haarlem, The Netherlands; m Department of Surgery, Franciscus Gasthuis & Vlietland, Rotterdam, The Netherlands; n Department of Surgery, Rijnstate Hospital, Arnhem, The Netherlands; o Department of Surgery, Zaans Medical Center, Zaandam, The Netherlands; p Department of Orthopaedic Surgery, Groene Hart Hospital, Gouda, The Netherlands; q Department of Trauma Surgery, Maastricht University Medical Center, Maastricht, The Netherlands; r Department of Orthopaedic Surgery, OLVG, Amsterdam, The Netherlands; s Department of Surgery, Albert Schweitzer Hospital, Dordrecht, The Netherlands; t Department of Surgery, Alrijne Hospital, Leiderdorp, The Netherlands; u Department of Surgery, Deventer Hospital, Deventer, The Netherlands; v Department of Surgery, Maasstad Hospital, Rotterdam, The Netherlands; w Department of Surgery, Elkerliek Hospital, Helmond, The Netherlands; x Department of Surgery, Franciscus Gasthuis & Vlietland, Schiedam, The Netherlands; y Department of Orthopaedic Surgery, IJsselland Hospital, Capelle a/d IJssel, The Netherlands; z Department of Surgery, Reinier de Graaf Gasthuis, Delft, The Netherlands; aa Department of Surgery, Elisabeth-TweeSteden Hospital, Tilburg, The Netherlands; ab Department of Surgery, Groene Hart Hospital, Gouda, The Netherlands; ac Department of Surgery, Hospital Gelderse Vallei, Ede, The Netherlands; ad Department of Orthopaedic Surgery, Deventer Hospital, Deventer, The Netherlands; ae Department of Surgery, Diakonessenhuis, Utrecht, The Netherlands; af Department of Surgery, Amphia Hospital, Breda, The Netherlands; ag Department of Surgery, Admiraal De Ruyter Hospital, Goes, The Netherlands; ah Department of Surgery, St. Antonius Hospital, Nieuwegein, The Netherlands; ai Department of Trauma Surgery, Amsterdam University Medical Center, location VUmc, Amsterdam, The Netherlands; Trauma Research Unit, Department of Surgery, Erasmus MC, University Medical Center Rotterdam, Rotterdam, The Netherlands

**Keywords:** Fracture, Humerus, Nonoperative, Operative, Radial nerve palsy, Shaft

## Abstract

**Background:**

The consequences of radial nerve palsy associated with a humeral shaft fracture are unclear. The aim of this study was to examine the functional recovery of radial nerve palsy, at presentation or postoperatively, in patients with a humeral shaft fracture.

**Methods:**

Data from patients who participated in the HUMeral shaft fractures: measuring recovery after operative versus non-operative treatment (HUMMER) study, a multicenter prospective cohort study including adults with a closed humeral shaft fracture Arbeitsgemeinschaft für Osteosynthesefragen (AO) type 12A or 12B, and had radial nerve palsy at presentation or postoperatively, were extracted from the HUMMER database. The primary outcome measure was clinically assessed recovery of motor function of the radial nerve. Secondary outcomes consisted of treatment, functional outcome (Disabilities of the Arm, Shoulder, and Hand and Constant–Murley Score), pain level, quality of life (Short Form-36 and EuroQoL-5D-3L), activity resumption, and range of motion of the shoulder and elbow joint at 12 months after trauma.

**Results:**

Three of the 145 nonoperatively treated patients had radial nerve palsy at presentation. One recovered spontaneously and 1 after osteosynthesis. Despite multiple surgical interventions, the third patient had no recovery after entrapment between fracture fragments. Thirteen of the 245 operatively treated patients had radial nerve palsy at presentation; all recovered. Nine other patients had postoperative radial nerve palsy; 8 recovered. One had ongoing recovery at the last follow-up, after nerve release and suture repair due to entrapment under the plate. At 12 months, the functional outcome scores of all patients suggested full recovery regarding functional outcome, pain, quality of life, activity resumption, and range of motion.

**Conclusion:**

Radial nerve palsy in patients with a humeral shaft fracture at presentation or postoperatively functionally recovers in 94% and 89%, respectively.

Radial nerve palsy is associated with humeral shaft fractures, whether primary due to the initial trauma or secondary as a consequence of treatment.^5^^,^^8^^,^^11^^,^^13^^,^^15^^,^^19^^,^^24^^,^^31^ The radial nerve is at risk due to its complex course, winding around the humeral shaft, and its close relationship to surrounding structures.^8^^,^^11^^,^^15^^,^^19^^,^^31^ As the radial nerve provides motor and sensory function to the arm, nerve damage can result in inability to extend and stabilize the wrist, also known as a wrist drop. Damage to the radial nerve causes difficulties in daily life as it severely compromises function and hand use.^15^^,^^21^

The reported rate of radial nerve palsy at presentation is approximately 10%.^11^ Reported rates of postoperative radial nerve palsy range from 3%-7%.^2^^,^^5^^,^^11^^,^^29^ Postoperative radial nerve palsy can be caused by manipulation and reposition, leading to neurapraxia, entrapment in the fracture site or compression by hardware, causing severe partial or complete lesions.^15^ Even though plate osteosynthesis with open reduction and internal fixation allows for direct visualization of the radial nerve, the implant placement, soft tissue preparation, and intraoperative nerve exploration increase the risk of iatrogenic radial nerve damage.^5^^,^^20^ Inherent to the treatment with intramedullary nailing (IMN), a risk of injuring the radial nerve arises due to manipulation of the fracture and the placement of distal screws nearby the radial nerve’s circuitous course around the distal humeral bone.^10^^,^^16^^,^^20^^,^^27^^,^^32^ A literature review, comparing plate osteosynthesis and IMN, has found similar rates of postoperative radial nerve palsy in both treatments.^35^

The influence of an existing or potential radial nerve palsy on the choice of the treatment of a humeral shaft fracture is not straightforward. The majority of palsies (88%-100%) will recover spontaneously in weeks to months after trauma.^2^^,^^11^^,^^23^ Therefore, Bishop and Ring concluded that there is no reason to solely operate on closed humeral shaft fractures because radial nerve palsy is present after trauma, and clinical monitoring is initially the best option.^3^ If signs of nerve recovery remain absent (after 4 months) or ultrasonography shows nerve damage, treatment is indicated. This can either be done with nonoperative treatment, such as bracing, rehabilitation, and electrostimulation, or surgical treatment, consisting of exploration, suture repair, and nerve and tendon transfer.^15^^,^^22^^,^^31^ However, the optimal treatment of radial nerve palsy and its influence on the choice of treatment of a humeral shaft fracture is currently controversial in clinical practice.

This prospective multicenter case series was performed as a secondary analysis to a large prospective cohort study of 390 patients with a closed humeral shaft fracture and reflects routine clinical practice. The aim of this study was to examine the consequences of a radial nerve palsy, at presentation and postoperatively, for patients with a closed humeral shaft fracture in terms of recovery and functional outcome in routine clinical practice.

## Methods

### Setting and participants

This case series was performed as a secondary analysis of the HUMeral shaft fractures: measuring recovery after operative versus non-operative treatment (HUMMER) study, a multicenter prospective cohort study conducted at 29 hospitals. The study design, methods, and primary outcome have been reported elsewhere.^7^^,^^17^ The HUMMER study was exempted by the local Medical Research Ethics Committee (no. MEC-2012-296) and recruited patients between October 23, 2012, and October 03, 2018. The Strengthening the Reporting of Observational Studies in Epidemiology (STROBE) guidelines for reporting of observational studies were followed.^30^ All patients gave written informed consent.

All patients aged 18 years or older with a closed humeral shaft fracture (Arbeitsgemeinschaft für Osteosynthesefragen [AO] type 12A or 12B; confirmed on X-ray) included in the HUMMER study, who either had radial nerve palsy at presentation or postoperatively, were included in this case series.

### Assessments and follow-up

Baseline patient characteristics (ie, age, gender, and dominance of the affected arm) and injury-related variables known to be associated with radial nerve palsy (ie, mechanism of injury, fracture location, and classification (according to the AO/Orthopaedic Trauma Association classification system) were extracted.^9^ The approach of fracture reduction (open or closed) and choice of treatment of the humeral shaft fracture was left up to the treating physician and was not dictated by the presence of radial nerve palsy at presentation.

The primary outcome measure was clinically assessed recovery of the radial nerve at 12 months follow-up. Recovery was defined as full recovery of motor function, including grip strength and wrist extension. Recovery of the radial nerve palsy was recorded during follow-up in the HUMMER study and based upon documented clinical assessment of recovery of motor function, as mentioned in the Dutch guidelines.^28^

Secondary outcomes extracted were the Disabilities of the Arm, Shoulder, and Hand (DASH) (ranging from 0-100 points, with a lower score representing less disability) and the Constant–Murley Score (ranging from 0-100 points, with a higher score representing better outcome) at 12 months follow-up.^1^^,^^6^^,^^12^ Furthermore, pain (Visual analog scale [VAS]; ranging from 0-10 points, with a higher score representing more pain), health-related quality of life (Short Form-36 [SF-36] and EuroQoL-5D-3L [EQ-5D-3L], with a higher score representing better quality of life), activity resumption (Numeric Rating Scale [NRS]; the extent to which patients resumed their activities at the pretrauma level), and range of motion of the shoulder and elbow joints, at 12 months follow-up were extracted.^4^^,^^33^^,^^34^ Quality of life scores were compared with published population norms (EQ-5D) or standardized combined scores (SF-36, mean of 50 ± 10 standard deviation [SD]).^14^^,^^26^ Range of motion of the shoulder and elbow joint were compared with reference values.^25^

### Statistical analysis

Analyses were performed using the Statistical Package for the Social Sciences (SPSS) version 25 (SPSS, Armonk, NY, USA). Normality of continuous data was tested with the Shapiro–Wilk test. Descriptive statistics were used to report the data. Continuous data are shown as median and percentiles (P_25_-P_75_; nonparametric). Categorical data are reported as N (%). The rates of radial nerve palsy at presentation and postoperatively are reported with 95% confidence intervals (95% CI). The secondary outcomes were extracted from the HUMMER database after comparison between treatment groups using linear mixed-effects regression models, as described before.^7^

## Results

### Patient and injury characteristics

**Figure 1:**
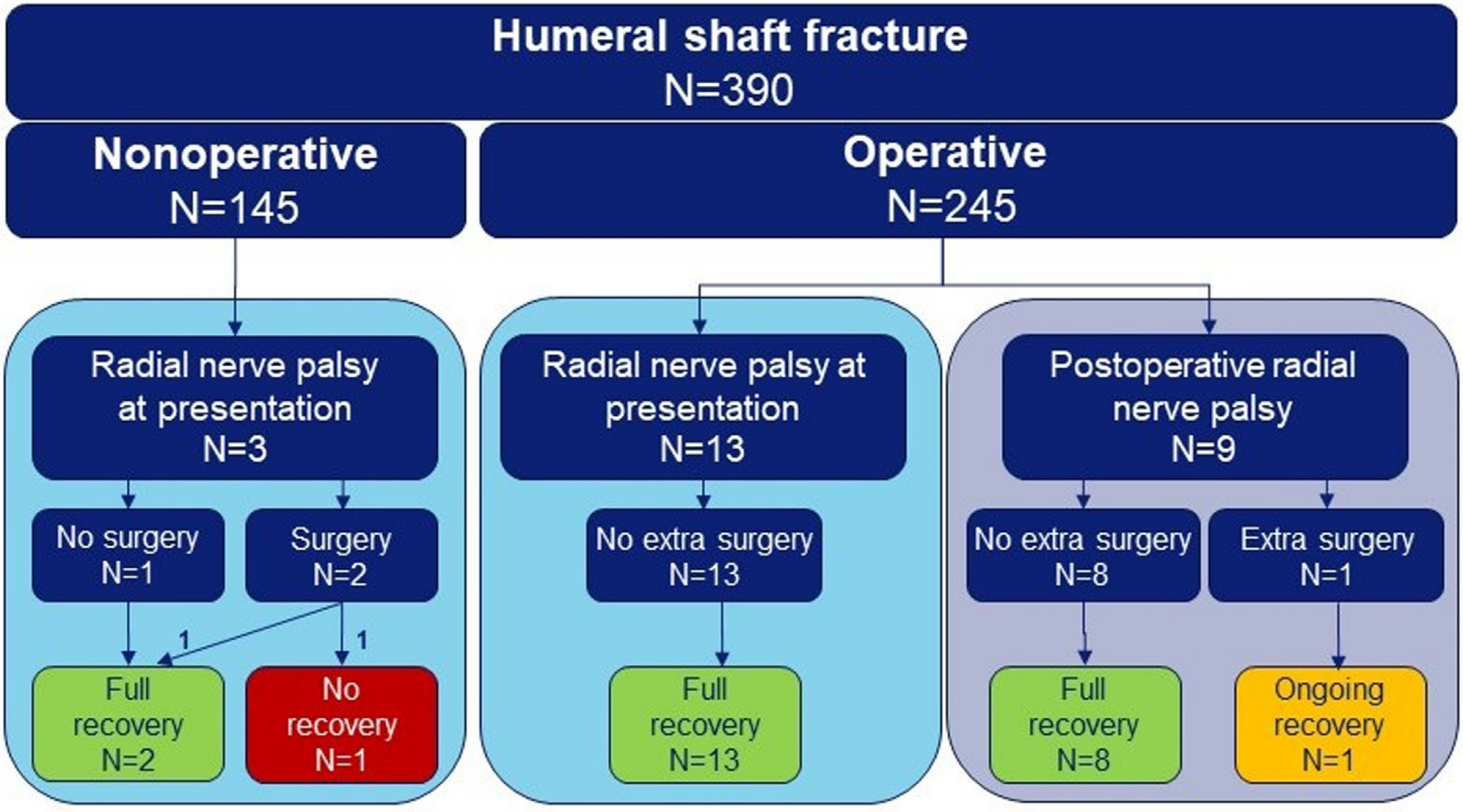
Flowchart of patients with radial nerve palsy in the study.

Twenty-five patients with a radial nerve palsy were included (Fig. 1 and Table I). Three patients were lost to follow-up, however, clinical documentation of treatment and recovery was retrieved locally. Out of the 390 patients, 16 (4.1% [95% CI 2.4-6.6]) presented with radial nerve palsy after trauma, of whom 13 were operated for their humeral shaft fracture. The group of patients with radial nerve palsy at presentation consisted of 9 men (56%) and had a median age of 49 years (P_25_-P_75_ 36-61). The mechanism of injury was frequently low energy trauma (N = 11; 69%). The fractures were often spiral (N = 8; 50%) and most often located in the middle of the humeral shaft (N = 14; 88%).

**Table I:** Patient, injury, treatment, and recovery details of radial nerve palsy in study participants.

Patient	Moment of diagnosis	Age (year)	Sex	AO classification	Location (third)	Dominant arm affected	Trauma mechanism	Treatment	Nerve identification	Macroscopic nerve lesion	Treatment of radial nerve palsy	Recovery 12 mo
1	Presentation	61	M	A1	Middle	Yes	HET	Brace	N.A.	N.A.	Secondary osteosynthesis (plate), Brace (cock-up)	Full
2	Presentation	69	M	A1	Middle	Yes	LET	Brace	N.A.	N.A.	Secondary osteosynthesis (IMN), nerve graft, brace (cock-up), hand therapy (N = 40), tendon transfer	No
3	Presentation	33	M	B1	Middle	Yes	LET	Brace	N.A.	N.A.	None	Full
4	Presentation	63	F	A1	Middle	Yes	LET	IMN	N.A.	N.A.	None	Full
5	Presentation	42	M	B2	Middle	No	HET	IMN	N.A.	N.A.	None	Full
6	Presentation	74	M	A3	Middle	Yes	LET	IMN	N.A.	N.A.	None	Full
7	Presentation	52	F	A1	Middle	No	LET	Plate	Yes	No	None	Full
8	Presentation	31	F	A2	Middle	Yes	LET	Plate	Yes	No	None	Full
9	Presentation	34	F	A3	Middle	No	LET	Plate	Yes	Partial	None	Full
10	Presentation	44	F	B2	Middle	No	HET	Plate	Yes	No	None	Full
11	Presentation	53	F	B1	Distal	Yes	LET	Plate	Yes	Partial	Brace (cock-up)	Full
12	Presentation	61	M	A3	Middle	No	LET	Plate	Yes	No	None	Full
13	Presentation	20	M	B1	Distal	No	HET	Plate	Yes	No	None	Full
14	Presentation	40	M	B1	Middle	Yes	LET	Plate	Yes	No	Brace (cock-up)	Full
15	Presentation	59	M	A2	Middle	Yes	LET	Plate	No	N.A.	None	Full
16	Presentation	46	F	A3	Middle	Yes	HET	Plate	Yes	No	Brace (cock-up), hand therapy (N = 13)	Full
17	Postoperative	57	M	A3	Middle	Yes	HET	IMN	No	N.A.	None	Full
18	Postoperative	65	F	B1	Middle	No	LET	Plate	Yes	Partial	None	Full
19	Postoperative	25	M	B1	Distal	Yes	LET	Plate	Yes	No	Brace (cock-up)	Full
20	Postoperative	32	F	A1	Distal	No	LET	Plate	No	N.A.	None	Full
21	Postoperative	30	M	B1	Distal	Yes	LET	Plate	Yes	Partial	Brace (cock-up)	Full
22	Postoperative	32	F	A1	Distal	No	LET	Plate	Yes	No	Brace (cock-up)	Full
23	Postoperative	62	M	A1	Middle	Yes	LET	Plate	Yes	Partial	Brace (cock-up)	Full
24	Postoperative	31	M	B2	Middle	Yes	HET	Plate	Yes	No	Brace (cock-up), hand therapy (N = 6)	Full
25	Postoperative	63	F	B3	Proximal	No	LET	Plate	No	N.A.	Nerve suture repair, brace (cock-up)	Ongoing

*AO*, Arbeitsgemeinschaft für Osteosynthesefragen; *F*, Female; *HET*, High energy trauma; *IMN*, Intramedullary nailing; *LET*, Low energy trauma; *M*, Male; *Mo*, months.

In 13 of the 245 operatively treated patients, postoperative radial nerve palsy could not be assessed, as they were already diagnosed with radial nerve palsy at presentation. Nine out of the remaining 232 (3.9% [95% CI 1.8-7.2]) operatively treated patients showed a postoperative radial nerve palsy, of which 5 men (56%) and a median age of 32 years (P_25_-P_75_ 30-63). Eight (89%) of these patients were treated with plate osteosynthesis and 1 (11%) with IMN. The mechanism of injury was frequently low energy trauma (N = 7; 78%). Six (67%) out of 9 patients had a spiral fracture. The fractures were located in the distal (N = 4; 44%), middle (N = 4; 44%), and proximal (N = 1; 12%) third of the humeral shaft.

### Treatment and recovery of radial nerve palsy at presentation

Three nonoperatively treated patients had radial nerve palsy at presentation, of whom 2 (67%) recovered. One (33%) recovered spontaneously. The other one (33%) recovered after secondary osteosynthesis with open plating 16 days post-trauma, with reported identification of an intact radial nerve, and postoperative treatment with a cock-up splint. The third (33%) patient did not regain radial nerve function. A secondary osteosynthesis with a retrograde IMN, 18 days post-trauma, without identification of the radial nerve was performed. An explorative revision surgery, 71 days post-trauma, showed a crushed radial nerve entrapped between fracture fragments. Subsequent nerve grafting, 7 months post-trauma, did not result in signs of improvement of function and further treatment including (cock-up) bracing and hand therapy, did not result in recovery of the radial nerve function either. The following tendon transfer also failed to restore wrist extension.

Ten (77%) of the 13 operatively treated patients with radial nerve palsy at presentation were treated with plate osteosynthesis and 3 (23%) with IMN. During surgery, the radial nerve was reported as identified in 9 (69%) out of the 13 patients. The identified radial nerve showed no macroscopic damage in 7 cases (77%) and a partial nerve lesion due to trauma in 2 cases (23%). Lesions were not addressed at the time of surgery. All operatively treated patients with radial nerve palsy at presentation spontaneously recovered after monitoring (N = 10; 77%) or treatment with a brace (cock-up; N = 3; 23%) or hand therapy (N = 1; 8%).

### Treatment and recovery of postoperative radial nerve palsy

Eight (89%) of the 9 patients with postoperative radial nerve palsy were treated for their humeral shaft fracture with plate osteosynthesis and 1(11%) with IMN. During surgery in 6 (67%) patients, the radial nerve was reported as identified and a partial macroscopic lesion was reported in 3 (50%) patients. The possible cause of the lesions was unknown. Lesions were not addressed at the time of surgery.

Postoperative radial nerve palsy recovered spontaneously without an additional surgical intervention for the nerve in 8 (89%) patients. Three (33%) patients were solely monitored and 5 (56%) were treated nonoperatively with bracing (cock-up; N = 6) or rehabilitation (hand therapy; N = 1). Absence of full recovery of postoperative radial nerve palsy occurred in 1 (11%) patient, after plate osteosynthesis with a Philos plate without identification of the radial nerve. An explorative revision surgery, performed 2 days later, indicated nerve release and suture repair due to entrapment under the plate. This resulted in signs of improvement and ongoing recovery at the last follow-up.

### Functional outcome after radial nerve palsy

At 12 months, the mean levels of functional outcome scores of patients with a radial nerve palsy, either at presentation or postoperatively, suggested full functional recovery regarding arm function (median DASH 8.3 [P_25_-P_75_ 7.4-11.1] and Constant–Murley 74 [P_25_-P_75_ 72-78]; Table II). Mean pain score was 1 (P_25_-P_75_ 1-2) and activities were resumed at pretrauma level (mean NRS of 9 [P_25_-P_75_ 9-9]). Health-related quality of life measured with the EuroQoL-5D-Utility Score (EQ-5D-US; 0.87 [P_25_-P_75_ 0.85-0.90] and EQ-5D-VAS 81 [P_25_-P_75_ 79-83]) were similar to the population norms (EQ-5D-US 0.89 and EQ-5D-VAS 81). The SF-36 scores (SF-36 Physical Component Summary [PCS] 50 [P_25_-P_75_ 48-52], SF-36 Mental Component Summary [MCS] 55 [P_25_-P_75_ 55-57]) were comparable with the standardized combined scores (SF-36 PCS 50; SF-36 MCS 50). Furthermore, functional levels of range of motion were achieved.

**Table II:** Functional outcome and range of motion of patients with radial nerve palsy at 12 months after trauma.

	All (N = 25)	Nonoperative treatment (N = 3)	Operative treatment (N = 22)	
		Radial nerve palsy at presentation (N = 3)	Radial nerve palsy at presentation (N = 13)	Postoperative radial nerve palsy (N = 9)
DASH	8.3 (7.4-11.1)	7.3 (2.5-8.7)	9.5 (7.8-11.7)	8.2 (5.7-11.8)
Constant–Murley	74 (72-78)	76 (74-82)	73 (70-77)	76 (71-80)
Pain (VAS)	1 (1-2)	1 (0-1)	1 (1-2)	1 (1-1)
Activity resumption (NRS)	9 (9-9)	9 (9-10)	9 (9-9)	9 (9-9)
SF-36 PCS	50 (48-52)	50 (49-54)	50 (48-51)	51 (48-52)
SF-36 MCS	55 (55-57)	55 (55-55)	55 (55-57)	56 (54-57)
EQ-5D-US	0.87 (0.85-0.90)	0.89 (0.87-0.93)	0.87 (0.85-0.89)	0.88 (0.84-0.91)
EQ-5D-VAS	81 (79-83)	78 (77-83)	81 (79-83)	81 (80-84)
Shoulder abduction (º)	138 (132-154)	138 (132-155)	136 (132-148)	143 (131-156)
Shoulder anteflexion (º)	140 (135-154)	140 (135-153)	138 (135-148)	145 (132-156)
Shoulder exorotation (º)	67 (64-73)	64 (62-69)	67 (65-71)	73 (63-74)
Shoulder endorotation (º)	63 (59-68)	59 (57-68)	65 (60-70)	63 (59-68)
Elbow flexion (º)	138 (137-139)	137 (135-137)	139 (138-140)	138 (137-140)
Elbow extension (º)	0 (0-2)	−4 (-4-0)	1 (0-3)	1 (0-2)
Elbow pronation (º)	85 (84-86)	82 (81-84)	85 (84-86)	85 (84-86)
Elbow supination (º)	83 (82-86)	80 (78-83)	84 (82-87)	84 (82-86)

*DASH*, Disabilities of the Arm, Shoulder, and Hand; *EQ-5D*, EuroQoL-5D; *MCS*, Mental Component Summary; *NRS*, Numerical Rating Scale; *PCS*, Physical Component Summary; *SF-36*, Short Form-36; *US*, Utility Score; *VAS*, Visual Analog Scale.

Data are presented as median (P_25_-P_75_).

The Constant–Murley Score, pain score, and ranges of motion of the shoulder and elbow joint are shown for the affected side.

## Discussion

The results of this study indicate that almost all radial nerve palsies spontaneously reach full recovery and the rate of persistent complaints due to radial nerve palsy at presentation in the HUMMER study is 0.3% (N = 1, ie, 1/390) and due to postoperative radial nerve palsy is 0.4% (N = 1, ie, 1/232). This study reports lower rates of radial nerve palsy at presentation (4.1% [95% CI 2.4-6.6]) than previously reported in a similar population (10%, ie, 88/922).^11^ Postoperative radial nerve palsy rates (3.9% [95% CI 1.8-7.2]) were similar as reported previously (ranging from 3%-7%).^5^^,^^11^^,^^29^ Recovery rates of radial nerve palsy at presentation (N = 15; 94%) and postoperatively (N = 8; 89%) were comparable with earlier cited literature (94% and 94%, respectively).^11^

Even though a higher DASH score may be expected as specific upper extremity functionalities rated in the DASH may be compromised if patients experience loss of extension due to radial nerve palsy, the DASH scores of patients with radial nerve palsy (8.3), were comparable with the level of all HUMMER patients at the 12-month follow-up (11.0 for the nonoperative and 8.8 for the operative group).^7^ Furthermore, the Constant–Murley Score, pain, activity resumption, and health-related quality of life scores were similar to those of the whole patient group, even though wrist drop can impact multiple aspects of these measures.^7^ All in all, the minimal risk of an impaired radial nerve function should be explained in shared decision making; however, it should be stressed that this is most often temporary.

Considering range of motion, a possible difference was expected in elbow extension and supination, as these movements are initiated by muscles (partly) innervated by the motor branch of the radial nerve (distal of a humeral shaft fracture; m. anconeus, m. brachialis, m. extensor carpi radialis longus, and m. supinator). However, the patients with radial nerve palsy achieved functional levels of range of motion, if compared with reference values and all HUMMER patients, suggesting that radial nerve palsy did not affect range of motion or disability was compensated by other muscles (eg, m. triceps for elbow extension and m. biceps for supination).^7^^,^^25^ In future research, range of motion of the wrist (flexion, extension, radial deviation, and ulnar deviation) should be assessed to examine all motor functions of the radial nerve.

This current data suggest that radial nerve palsy at presentation is no indication for operative exploration as almost all palsies recovered spontaneously without a secondary intervention. The HUMMER study showed that there was no tendency to treat patients with radial nerve palsy at presentation operatively.^7^ Nerve identification during secondary surgical procedures showed very few partial and no complete macroscopic lesions of the radial nerve, suggesting that radial nerve palsy is mostly caused by temporary neurapraxia. However, if entrapment of the nerve by fracture fragments is suspected, the use of ultrasound as a diagnostic modality is indicated, given its noninvasive nature and its ability to accurately diagnose entrapment or lesions of the radial nerve with a sensitivity and specificity of 89% and 95%, respectively.^18^ In case of entrapment or lesions, immediate nerve exploration, release and suture repair is indicated to allow for recovery of nerve function.

It should be conveyed that, since postoperative radial nerve palsy is rare and almost always spontaneously recovers, persistent postoperative radial nerve palsy should be no discouragement for operative treatment of humeral shaft fractures. However, safe surgical procedures are only possible with careful nerve exploration and identification, which is most feasible during open plate osteosynthesis. Written and visual confirmation of the safe position of the radial nerve relative to the implant are desired to facilitate shared decision making in the case of persistent palsy in order to rule out the possibility of entrapment. Only if radial nerve palsy is persistent, surgical documentation is incomplete, and ultrasound implies a complete lesion or entrapment, secondary surgical exploration is indicated.

### Strengths and limitations

The main strength of this case series is that the prospective design allows for generalizable and clinically relevant results. A limitation of this study is that the study design did not include a protocol for the assessment and treatment of radial nerve palsy, resulting in heterogeneity in the choice of diagnostic instruments and management strategies. Since years of experience are not included in the HUMMER database, it is unclear if the occurrence of iatrogenic radial nerve palsy in operatively treated patients could be attributed to experience of the surgeon. Furthermore, the relatively low number of cases can be critiqued, however, cannot be avoided due to the low prevalence of radial nerve palsy associated with humeral shaft fractures.

## Conclusions

Radial nerve palsy in patients with a humeral shaft fracture at presentation or postoperatively functionally recovers in 94% and 89%, respectively.

## Disclaimers

Funding: This project was supported by a grant from the 10.13039/100009363Osteosynthesis and Trauma Care (OTC) Foundation (reference number 2013-DHEL). This organization was not involved in the study design, patient recruitment, data collection, data analysis, data interpretation, publication decisions, or in any aspect pertinent to this study.

Conflicts of interest: DDH and EMMVL had financial support from the OTC Foundation for the submitted work; no financial relationships with any organizations that might have an interest in the submitted work in the previous 3 years; no other relationships or activities that could appear to have influenced the submitted work. The other authors, including the HUMMER investigators, their immediate families, and any research foundation with which they are affiliated have not received any financial payments or other benefits from any commercial entity related to the subject of this article.

## Appendix. HUMMER Investigators

Local principal investigators and co-investigators. Ivo Beetz, MD, PhD, Trauma Research Unit Department of Surgery, Erasmus MC, University Medical Center Rotterdam, Rotterdam, The Netherlands; Hugo W. Bolhuis, MD, Department of Surgery, Gelre Hospital, Apeldoorn, The Netherlands; P. Koen Bos, MD, PhD, Department of Orthopaedic Surgery, Erasmus MC, University Medical Center Rotterdam, Rotterdam, The Netherlands; Maarten W. G. A. Bronkhorst, MD, PhD, Trauma Unit, Haaglanden MC, The Hague, The Netherlands; Milko M. M. Bruijninckx, MD, Department of Surgery, IJsselland Hospital, Capelle a/d Ijssel, The Netherlands; Jeroen De Haan, MD, PhD, Department of Surgery, Dijklander Ziekenhuis, Hoorn, The Netherlands; Axel R. Deenik, MD, PhD, Department of Orthopaedic Surgery, Haaglanden MC, The Hague, The Netherlands; P. Ted Den Hoed, MD, PhD, Department of Surgery, Ikazia Hospital, Rotterdam, The Netherlands; Martin G. Eversdijk, MD, Department of Surgery, St. Jansdal Hospital, Harderwijk, The Netherlands; J. Carel Goslings, MD, PhD, Trauma Unit, Department of Surgery, Amsterdam University Medical Center, location AMC, Amsterdam, The Netherlands; Robert Haverlag, MD, Department of Surgery, OLVG, Amsterdam, The Netherlands; Martin J. Heetveld, MD, PhD, Department of Surgery, Spaarne Gasthuis, Haarlem, The Netherlands; Albertus J. H. Kerver, MD, PhD, Department of Surgery, Franciscus Gasthuis & Vlietland, Rotterdam, The Netherlands; Karel A. Kolkman, MD, Department of Surgery, Rijnstate Hospital, Arnhem, The Netherlands; Peter A. Leenhouts, MD, MBA, Department of Surgery, Zaans Medical Center, Zaandam, The Netherlands; Kiran C. Mahabier, MD, PhD, Trauma Research Unit, Department of Surgery, Erasmus MC, University Medical Center Rotterdam, Rotterdam, The Netherlands; Sven A. G. Meylaerts, MD, PhD, Trauma Unit, Haaglanden MC, The Hague, The Netherlands; Ron Onstenk, MD, Department of Orthopaedic Surgery, Groene Hart Hospital, Gouda, The Netherlands; Martijn Poeze, MD, PhD, Department of Trauma Surgery, Maastricht University Medical Center, Maastricht, The Netherlands; Rudolf W. Poolman, MD, PhD, Department of Orthopaedic Surgery, OLVG, Amsterdam, The Netherlands; Bas J. Punt, MD, Department of Surgery, Albert Schweitzer Hospital, Dordrecht, The Netherlands; Ewan D. Ritchie, MD, Department of Surgery, Alrijne Hospital, Leiderdorp, The Netherlands; W. Herbert Roerdink, MD, PhD, Department of Surgery, Deventer Hospital, Deventer, The Netherlands; Gert R. Roukema, MD, Department of Surgery, Maasstad Hospital, Rotterdam, The Netherlands; Jan Bernard Sintenie, MD, Department of Surgery, Elkerliek Hospital, Helmond, The Netherlands; Nicolaj M. R. Soesman, MD, Department of Surgery, Franciscus Gasthuis & Vlietland, Schiedam, The Netherlands; Edgar J. T. Ten Holder, MD, Department of Orthopaedic Surgery, IJsselland Hospital, Capelle a/d IJssel, The Netherlands; Wim E. Tuinebreijer, MD, PhD, Trauma Research Unit, Department of Surgery, Erasmus MC, University Medical Center Rotterdam, Rotterdam, The Netherlands; Maarten Van der Elst, MD, PhD, Department of Surgery, Reinier de Graaf Gasthuis, Delft, The Netherlands; Frank H. W. M. Van der Heijden, MD, PhD, Department of Surgery, Elisabeth-TweeSteden Hospital, Tilburg, The Netherlands; Frits M. Van der Linden, MD, Department of Surgery, Groene Hart Hospital, Gouda, The Netherlands; Peer Van der Zwaal, MD, PhD, Trauma Unit, Haaglanden MC, The Hague, The Netherlands; Jan P. Van Dijk, MD, Department of Surgery, Hospital Gelderse Vallei, Ede, The Netherlands; Hans-Peter W. Van Jonbergen, MD, PhD, Department of Orthopaedic Surgery, Deventer Hospital, The Netherlands; Egbert J. M. M. Verleisdonk, MD, PhD, Department of Surgery, Diakonessenhuis, Utrecht, The Netherlands; Jos P. A. M. Vroemen, MD, PhD, Department of Surgery, Amphia Hospital, Breda, The Netherlands; Marco Waleboer, MD, Department of Surgery, Admiraal De Ruyter Hospital, Goes, The Netherlands; Philippe Wittich, MD, PhD, Department of Surgery, St. Antonius Hospital, Nieuwegein, The Netherlands; Wietse P. Zuidema, MD, Department of Trauma Surgery, Amsterdam University Medical Center, location VUmc, Amsterdam, The Netherlands. Medical students (Trauma Research Unit, Department of Surgery, Erasmus MC, University Medical Center Rotterdam, Rotterdam, The Netherlands): Ahmed Al Khanim; Jelle E. Bousema; Kevin Cheng; Yordy Claes; J. Daniël Cnossen; Emmelie N. Dekker; Aron J. M. De Zwart; Priscilla A. Jawahier; Boudijn S. H. Joling; Cornelia (Marije) A. W. Notenboom; Jaap B. Schulte; Nina Theyskens; Gijs J. J. Van Aert; Boyd C. P. Van der Schaaf; Tim Van der Torre; Joyce Van Veldhuizen; Lois M. M. Verhagen; Maarten Verwer; Joris Vollbrandt.

## References

[1] Beaton D.E., Katz J.N., Fossel A.H., Wright J.G., Tarasuk V., Bombardier C. (2001). Measuring the whole or the parts? Validity, reliability, and responsiveness of the disabilities of the arm, shoulder and hand outcome measure in different regions of the upper extremity. J Hand Ther.

[2] Belayneh R., Lott A., Haglin J., Konda S., Leucht P., Egol K. (2019). Final outcomes of radial nerve palsy associated with humeral shaft fracture and nonunion. J Orthop Traumatol.

[3] Bishop J., Ring D. (2009). Management of radial nerve palsy associated with humeral shaft fracture: a decision analysis model. J Hand Surg Am.

[4] Brooks R., Rabin R.E., de Charro F. (2003). The measurement and valuation of health status using EQ-5D: A European perspective.

[5] Claessen F.M., Peters R.M., Verbeek D.O., Helfet D.L., Ring D. (2015). Factors associated with radial nerve palsy after operative treatment of diaphyseal humeral shaft fractures. J Shoulder Elbow Surg.

[6] Constant C.R., Murley A.H. (1987). A clinical method of functional assessment of the shoulder. Clin Orthop Relat Res.

[7] Den Hartog D., Van Bergen S.H., Mahabier K.C., Verhofstad M.H.J., Van Lieshout E.M.M., Investigators H. (2022). Functional and clinical outcome after operative versus nonoperative treatment of a humeral shaft fracture (HUMMER): results of a multicenter prospective cohort study. Eur J Trauma Emerg Surg.

[8] Ekholm R., Ponzer S., Tornkvist H., Adami J., Tidermark J. (2008). The Holstein-Lewis humeral shaft fracture: aspects of radial nerve injury, primary treatment, and outcome. J Orthop Trauma.

[9] (1996). Fracture and dislocation compendium. Orthopaedic Trauma Association Committee for Coding and Classification. J Orthop Trauma.

[10] Grass G., Kabir K., Ohse J., Rangger C., Besch L., Mathiak G. (2011). Primary exploration of radial nerve is not required for radial nerve palsy while treating humerus shaft fractures with unreamed humerus nails. Open Orthop J.

[11] Hendrickx L.A.M., Hilgersom N.F.J., Alkaduhimi H., Doornberg J.N., van den Bekerom M.P.J. (2021). Radial nerve palsy associated with closed humeral shaft fractures: a systematic review of 1758 patients. Arch Orthop Trauma Surg.

[12] Hudak P.L., Amadio P.C., Bombardier C. (1996). Development of an upper extremity outcome measure: the DASH (disabilities of the arm, shoulder and hand) [corrected]. The Upper Extremity Collaborative Group (UECG). Am J Ind Med.

[13] Im J.H., Moon D.K., Gwark J.Y., Park H.B. (2021). Need for early exploration of radial nerve in humeral shaft fractures with radial nerve palsy. Arch Orthop Trauma Surg.

[14] Lamers L.M., Stalmeier P.F., McDonnell J., Krabbe P.F.V.B., van Busschbach J.J. (2005). [Measuring the quality of life in economic evaluations: the Dutch EQ-5D tariff]. Ned Tijdschr Geneeskd.

[15] Laulan J. (2019). High radial nerve palsy. Hand Surg Rehabil.

[16] Lin J. (2002). Locked nailing of spiral humeral fractures with or without radial nerve entrapment. Clin Orthop Relat Res.

[17] Mahabier K.C., Van Lieshout E.M.M., Bolhuis H.W., Bos P.K., Bronkhorst M.W., Bruijninckx M.M. (2014). HUMeral shaft fractures: measuring recovery after operative versus non-operative treatment (HUMMER): a multicenter comparative observational study. BMC Musculoskelet Disord.

[18] Cartwright M.S., Chloros G.D., Walker F.O., Wiesler E.R., Campbell W.W. (2007). Diagnostic ultrasound for nerve transection. Muscle Nerve.

[19] Noaman H., Khalifa A.R., El-Deen M.A., Shiha A. (2008). Early surgical exploration of radial nerve injury associated with fracture shaft humerus. Microsurgery.

[20] Ostermann R.C., Lang N.W., Joestl J., Pauzenberger L., Tiefenboeck T.M., Platzer P. (2019). Fractures of the humeral shaft with primary radial nerve palsy: do injury mechanism, fracture type, or treatment influence nerve recovery?. J Clin Med.

[21] Ricci F.P., Barbosa R.I., Elui V.M., Barbieri C.H., Mazzer N., Fonseca Mde C. (2015). Radial nerve injury associated with humeral shaft fracture: a retrospective study. Acta Ortop Bras.

[22] Schwab T.R., Stillhard P.F., Schibli S., Furrer M., Sommer C. (2018). Radial nerve palsy in humeral shaft fractures with internal fixation: analysis of management and outcome. Eur J Trauma Emerg Surg.

[23] Shao Y.C., Harwood P., Grotz M.R., Limb D., Giannoudis P.V. (2005). Radial nerve palsy associated with fractures of the shaft of the humerus: a systematic review. J Bone Joint Surg Br.

[24] Shivarathre D.G., Dheerendra S.K., Bari A., Muddu B.N. (2008). Management of clinical radial nerve palsy with closed fracture shaft of humerus--a postal questionnaire survey. Surgeon.

[25] Soucie J.M., Wang C., Forsyth A., Funk S., Denny M., Roach K.E. (2011). Range of motion measurements: reference values and a database for comparison studies. Haemophilia.

[26] Szende A., Janssen B., Cabases J. (2014). Self-reported population health: An International perspective based on EQ-5D.

[27] Theeuwes H.P., van der Ende B., Potters J.W., Kerver A.J., Bessems J., Kleinrensink G.J. (2017). The course of the radial nerve in the distal humerus: a novel, anatomy based, radiographic assessment. PLoS One.

[28] Traumacentrum West (2016).

[29] Van de Wall B.J.M., Baumgartner R., Houwert R.M., Link B.C., Heng M., Knobe M. (2022). MIPO versus nailing for humeral shaft fractures: a meta-analysis and systematic review of randomised clinical trials and observational studies. Eur J Trauma Emerg Surg.

[30] Von Elm E., Altman D.G., Egger M., Pocock S.J., Gøtzsche P.C., Vandenbroucke J.P. (2014). The Strengthening the Reporting of Observational Studies in Epidemiology (STROBE) statement: guidelines for reporting observational studies. Int J Surg.

[31] Venouziou A.I., Dailiana Z.H., Varitimidis S.E., Hantes M.E., Gougoulias N.E., Malizos K.N. (2011). Radial nerve palsy associated with humeral shaft fracture. Is the energy of trauma a prognostic factor?. Injury.

[32] Wang J.P., Shen W.J., Chen W.M., Huang C.K., Shen Y.S., Chen T.H. (2009). Iatrogenic radial nerve palsy after operative management of humeral shaft fractures. J Trauma.

[33] Ware J.E., Gandek B., Kosinski M., Aaronson N.K., Apolone G., Brazier J. (1998). The equivalence of SF-36 summary health scores estimated using standard and country-specific algorithms in 10 countries: results from the IQOLA Project. International Quality of Life Assessment. J Clin Epidemiol.

[34] Ware J.E., Sherbourne C.D. (1992). The MOS 36-item short-form health survey (SF-36). I. Conceptual framework and item selection. Med Care.

[35] Zhao J.G., Wang J., Wang C., Kan S.L. (2015). Intramedullary nail versus plate fixation for humeral shaft fractures: a systematic review of overlapping meta-analyses. Medicine (Baltimore).

